# Transparency in healthcare AI: Testing EU regulatory provisions against users’ transparency needs

**DOI:** 10.1371/journal.pdig.0001594

**Published:** 2026-07-24

**Authors:** Anna Spagnolli, Cecilia Tolomini, Elisa Beretta, Patrik Pluchino, Claudio Sarra

**Affiliations:** 1 Dipartimento di Psicologia Generale, Università degli Studi di Padova, Padova, Italy; 2 Human Inspired Technologies Research Centre, Università degli Studi di Padova, Padova, Italy; 3 Dipartimento di Diritto Privato e Critica del Diritto, Università degli Studi di Padova, Padova, Italy; Yonsei University College of Medicine, KOREA, REPUBLIC OF

## Abstract

Algorithmic systems play an essential role in healthcare and are pervasively incorporated into medical software and equipment. In the European Union, providers must share transparency information with deployers (users) via a document called Instructions for Use (IFU, Directive 2024/1689 or AI Act). This study tests the extent to which deployers’ transparency needs map onto the structure of an IFU document. A survey was administered online via the Qualtrics platform to four types of deployers, i.e., managers (N = 238), healthcare professionals (N = 115), patients (N = 229), and information technology workers (N = 230). The participants rated the relevance of a set of transparency needs and selected the IFU section more likely to address them. The results reveal differentiated priorities across user types and reveal potential weaknesses in locating some transparency information. Recommendations to build a locally meaningful IFU are derived.

## Author summary

Healthcare “artificial intelligence” devices must include transparency information under the European Union law, including a list of specific information items. We wondered whether users orient themselves within that list, and if health professionals, patients, managers, and information technology workers have different transparency priorities. We asked 813 respondents to rate the relevance of 24 types of transparency information and to guess where to find them in the transparency documentation. We identified how transparency needs vary across user types, and which information is harder to find. We derive recommendations for designing transparency documentation.

## 1. Introduction

Algorithmic systems can analyze massive amounts of data to make inferences, recognize patterns, and draw predictions. In healthcare, these capabilities are applied to clinical practice to make predictions at the individual level combining patients’ medical histories, symptoms, and test results [1–8] organize a knowledge domain for patients [4] and health professionals [9], force steps to minimize human errors during decision-making [10], guide robotic equipment to enhance the precision of human movements during surgical operations [11] and analyze data from the operating field [12]. Telemedicine applications can support treatment programs with remote reminders, data collection, and analysis [1]. Healthcare facilities can adopt AI solutions to organize supply chains, revenue cycles, and patient scheduling [13] and support data analysis during clinical trials [4]. AI-based virtual and mixed reality simulations can be used to train medical students [2,5].

Recent regulations and guidelines, such as the EU AI Act [14], the US AI Risk Management Framework [15], or UNESCO’s recommendations on the Ethics of Artificial Intelligence [16], aim to enhance the trustworthiness of algorithmic systems. In the area of digital health, they combine with regulations for medical devices, such as [17,18] in the EU, to define what to include in the device’s technical documentation [19,20]. The AI Act establishes stringent and non-discretionary transparency obligations for providers of devices - such as healthcare ones [21] - whose malfunctioning can directly damage the users’ rights and well-being (high-risk devices). One obligation is to prepare a notice to users (or “deployers,” in regulatory terminology) to facilitate informed, responsible, and proper use of the technology. These Instructions for Use (IFU) must contain information on the systems’ capabilities, limitations, and security as described in Article 13 of the regulation.

The field of usable privacy [22] analyses compliance with privacy and regulatory provisions from the perspective of its usability, defined as “the extent to which a system, product or service can be used by specified users to achieve specified goals with effectiveness, efficiency and satisfaction in a specified context of use” [23]. Specifically, full compliance with the principle of transparency requires that the transparency information is understandable and relevant to users (“meaningful transparency” [24,25]).

In this study, we assess whether the IFU structure serves as an effective entry point for users seeking transparency information about healthcare devices equipped with AI solutions. We compiled a list of transparency needs from the literature and investigated if they map well onto the IFU sections listed in Article 13 of the AI Act. Participants were asked to locate the IFU section most likely to fulfill a given transparency need: the more dispersed a need across sections, the more ambiguous the mapping. Additionally, we measured the relevance of the transparency needs to our sample and subsamples. To our knowledge, no previous study has attempted to do so, except for Gils’ team [26], who prepared and tested IFU documents across several use cases, including health devices, involving 21 and 15 stakeholders. Our focus differs, and so does the scale of the sample involved, including 812 participants across four classes of potential users; nonetheless, some of Gil’s final recommendations align with our findings and are included in the discussion. The contributions of this study consist of (a) testing the IFU’s structure against transparency needs in healthcare AI; (b) providing design recommendations to improve transparency documentation responsiveness to such needs; (c) showcasing a methodology to evaluate the effectiveness of transparency documentation, which can be exported outside the health domain and scaled down to specific AI systems.

## 2. Method

Our method consists of outlining a list of user-based transparency needs, recruiting the different user types targeted by the documentation, and asking users to validate the needs and localize them within the IFU sections. The results identify the most likely IFU sections to accommodate specific transparency needs, assess the level of ambiguity in the structure itself, and validate the relevance of the transparency needs.

### 2.1 Ethics

The study was approved by the Ethical Committee of the Human Inspired Technology Research Centre at the University of Padova, Italy (approval number 2024_263R1). The written formal consent was obtained from all participants according to the procedure described in the Procedure section.

### 2.2 IFU structure

The IFU structure tested in the study is the simplified version of the 12 mandatory pieces of information described in the AI Act. We removed articles, repeated references to provider, deployer, or AI systems, adverbs expressing conditions (i.e., where relevant, if any, where applicable), non-informative modifiers or heads of modifiers (a description of…), and references to other articles of the Act. We also streamlined the structure by adding pre-modifications (e.g., “needed hardware resources” instead of “hardware resources needed”) and itemizations (e.g., bullet points). Lexical changes were minimal to avoid confusing respondents familiar with some of the terminology.

We also assigned each piece of information an icon. To our knowledge, there is no established set of validated icons for AI transparency compliance, apart from the work by Friederich and colleagues, which is highly specific to AI data [27] and on privacy icons [28], which do not apply to this case. Therefore, we relied on the Microsoft 365 icon library [29] and searched for semantically compatible icons that match the IFU content.

The result is the “Simplified IFU” structure, consisting of 12 section titles (Table 1). Its relationship with the original wording in the AI Act is reported in the Supporting Information (Table A in Zenodo archive [37]).

**Table 1:** The 12 sections of the IFU structure in this study.

Capabilities and limitations
1. Intended purpose of the AI system
2. Correctness, performance resilience and cybersecurity.
3. Risks to health, safety, or fundamental rights.
4. Capabilities to explain its results.
5. Performance with specific (groups of) persons.
6. Data sets used to train, validate and test the system
7. Proper usage
**Other information**
8. Provider’s identity, contact details and authorised representative
9. Collection, storage and interpretation of activities’ record
10. Needed computational and hardware resources, maintenance/care, expected lifetime
11. Planned changes to AI system
12. Human control over the machine, including support in interpreting the results

### 2.3 Transparency needs

The core part of the survey identified some transparency needs and asked participants to select the IFU section most likely to address them (Figure A in Zenodo archive [37]). The full list of transparency needs is reported in Table 2, organized into four groups by topic similarity. The list is adapted from Vo and colleagues [30], who systematically reviewed 105 studies on AI in healthcare that involved health professionals and patients. Each transparency need is prefaced by a possible concern followed by a related information need, for instance, “The AI device could be misused to alter or take advantage of health results. Are there measures in place to prevent this?”

**Table 2:** Transparency needs used in this study.

Group	Abbrev	Content
Accuracy	A - bs	The AI device might not have been tested on all patient types. What patient types are underrepresented?
Accuracy	a - cl	The AI device needs to perform its intended job reliably. Was it tested to ensure it meets expectations?
Accuracy	a - cm	Each medical situation is complex. What characteristics does the AI take into account of each medical situation?
Accuracy	a - pp	The AI might not suit all patients. Are there patients for which this device is not recommended?
Accuracy	a - pr pt	This AI device must have been in use long enough. On how many patients has it been used?
Accuracy	a - ps	I wonder what patients will feel. Were past patients happy with the care provided with the AI device?
Humanization	h - br	Language, culture, or financial barriers could make using the AI device challenging. What are the requirements to use it?
Humanization	h - cn	Human contact is important in clinical practice. Do doctors and patients still interact with each other when this AI device is used?
Humanization	h - ov	The AI device might malfunction or make mistakes. Is there a person overseeing the AI device?
Humanization	h - pr	Doctors have many tasks besides treatment, such as creating summary letters for patients. Does the AI device have features that assist in all aspects of their job?
Humanization	h - sp	Each patient is unique. Does the AI device consider the unique characteristics of each patient?
Humanization	h - un	I might not understand the AI output; does the device come with simple explanations of what its results mean?
Law	l - adv	Some digital devices include advertisements. Does this one have any?
Law	l - cy	Unauthorized outsiders might try to access the device; is it protected against that?
Law	l - dt	The AI device surely collects my personal data and health information. Does this AI device keep it protected?
Law	l - lb	There is always a chance that something can go wrong with a treatment. If something happens, is the responsibility for the mistake with the AI device or the user?
Law	l - ms	The AI device could be misused to alter or take advantage of health results. Are there measures in place to prevent this?
Law	l – l	For the device to be legal, it must follow certain regulations and official guidelines. What are they?
Resources	r - fn	The proper functioning of hardware and software, a stable connection, and access to technical support are crucial for the device’s reliability. What guarantees are there for these aspects?
Resources	r - fu	The AI equipment and research are expensive. Is there any funding to cover the high costs?
Resources	r - hl	The AI device should work with the healthcare system in my region. Is the AI device connected to it?
Resources	r - in	Treatments with this AI device seem unusual and fancy. Do insurance companies cover treatments that use it?
Resources	r - tr	I do not want to be left alone to figure out how to use the AI device. Is there any training available to help users operate it?
Resources	r - us	Ease of use is essential for any device. Was the system tested to ensure it is user-friendly?

### 2.4 Measures

The core part of the questionnaire deployed in our study was the transparency need block, which included a transparency need and three questions:

- Localizability of the need in the IFU sections; this item was a multiple-choice question asking to select the IFU section most likely to contain an answer to the transparency need. An open-ended question followed, asking the participant to motivate their selection (“Please explain the reasons for selecting that IFU section”). The open question was meant to encourage a pondered selection and was not analyzed.
- Confidence with the selection made, measured with the item “How confident are you that it is the right section?” on a 5-point scale from 5 (very confident) to 1 (very doubtful).
- Perceived relevance of the transparency needs, measured with the item “How relevant is that information to trustfully use an AI device?” on a 5-degree scale from 5 (absolutely relevant) to 1 (totally irrelevant).

#### 2.4.1 Platform.

The questionnaire was built and administered via the online survey platform Qualtrics [31]. No restrictions were placed on the device to be used, and the survey format was optimized for mobile devices according to Qualtrics guidelines (e.g., using a multiple-choice question format instead of matrices, limiting the number of answer columns to two, and aligning the answer options vertically). The questionnaire included a few strategies to maximize the quality of the data collected. To discourage rushed answers, the instruction pages and question blocks were timed so that participants could proceed only after a predefined number of seconds. An item prevented the participants who ignored the introductory part of the survey from continuing. It consisted of a multiple-choice question asking, “So, what is an IFU?” with three answer options: “A document with instructions for the users of an AI device” (correct), “An AI device supporting physicians in healthcare,” or “The name of a recent EU regulation regarding healthcare.” Only data from participants recognizing the correct answer were analyzed. The deactivation of multiple submissions from the same ID and the inclusion of a bot check (“Captcha”) prevented respondents from using software that automatically fills in forms [32]. Steps were also taken to prevent open-ended questions from being answered by LLMs: LLMs were explicitly forbidden in the instructions and made technically difficult by removing the ability to copy and paste text to and from the survey. This combination discourages the use of LLMs better than other strategies [33].

### 2.5 Procedure

The protocol started with the invitation sent by the recruitment platform to all eligible subscribers: “We are investigating the information needed to use an AI system trustfully. If you participate, you will rate the relevance of such information and locate it in the table of contents of the instructions sheet. We are very interested in your answers, so the survey includes attention checks and adopts technical deterrents against using LLM to write answers. Completing the survey and passing attention checks is compensated with 3 UK£.” The invitees interested in participating were directed to the online questionnaire, which opened with a short version of the information note. From there, they could download the complete, detailed note if they wished. Consent was expressed by pressing the “I agree” option at the bottom of the information note page.

Participants who agreed to participate proceeded to the questionnaire and were introduced to the study’s topic, the IFU. They were first provided a brief description (“Artificial Intelligence technologies can support healthcare professionals in tasks such as diagnosis, information retrieval, treatment decisions, surgical operations, and the analysis of large amounts of data during trials. A mandatory document called ‘IFU’ (‘Instructions for Use’) accompanies any AI device and addresses its users. Users can be hospital administrators, physicians, technicians, or patients. An IFU contains 12 sections.”). The IFU structure was then displayed, and the participants were asked to highlight the words that were obscure to them. They had no restrictions on the number of words that could be highlighted, including none (in this case, a confirmation message appeared to ensure that the lack of selection was deliberate). All words could be highlighted except articles, conjunctions, and prepositions. The purpose of this part was to help respondents familiarize themselves with the IFU, thereby improving the quality of the subsequently collected data. An attention check was then administered and marked as such to signal the need to answer that item carefully and avoid adverse reactions observed with hidden attention checks [34]. It read as follows: “So, what is an IFU? *[Answer options]*: A document with instructions for the users of an AI device *[correct]*/an AI device supporting physicians in healthcare*[wrong]*/the name of a recent EU regulation regarding healthcare *[wrong]*.” The order of appearance of the three options was randomized across the sample, so the correct option did not always appear first. Participants who provided an incorrect answer ended the survey there and were compensated with 0.5£ for having spent some time (median=3 minutes) in the survey, as recommended by the recruitment platform’s policy.

The participants then moved to the core part of the questionnaire and were given the following instructions: “We will show 2 questions the AI user might have. 1. Read the question. 2. Judge its relevance. 3. Select the IFU section possibly containing the answer. It takes 30 seconds before the second question is displayed to prevent rushed answers*.* Thank you in advance for your time on this project!*”* After 10 seconds had passed, a forward button became available, and the participants started the core part of the survey. Automated randomizers selected a transparency block, first choosing among four groups of needs available (i.e., accuracy, humanization, law, and resources) and then among the six needs in the group. The randomizers were set to display all needs the same number of times across each subsample (by selecting “evenly present elements” in Qualtrics randomizers). A single participant was only asked to identify two transparency needs to avoid careless answers dictated by fatigue. A timer was visible (counting up instead of down, to avoid suggesting a time limit). Once the two transparency blocks were completed, the participants were thanked, and the survey ended. Median completion time was 8,4 minutes in the health professionals subsample, 10 minutes in the other three subsamples.

### 2.6 Pilots

We surveyed 12 participants to test the data collection procedure and added an item to solicit feedback. Based on these pilots, we made some adjustments to the initial setup of the survey mechanism. The change inspired by the participant’s feedback consisted of autoloading all instructions within one instructions page instead of automatically progressing through several instruction pages, to accommodate every reading speed. We also randomized complete blocks instead of randomizing the transparency needs part only, because we realized that scores would not be associated with needs IDs in the Qualtrics dataset.

### 2.7 Data analysis

The data collected allowed us to calculate the following main measures:

- Selection frequency of an IFU section per transparency need;
- Confidence scores per selection;
- Relevance score per transparency need.

To test if a need was univocally mapped onto a given section, we considered the frequency with which all 12 sections were chosen for that need and assessed if each section had an equal probability of being picked with a Chi-Square test. To test whether the level of convergence on the IFU sections correlated with the participants’ confidence, we calculated the Spearman rank-order correlation between the proportion of respondents selecting the most frequently chosen section and the mean confidence score across the 24 transparency needs.

To validate the perceived relevance of the needs included in the study, we performed Wilcoxon signed-rank tests comparing each need’s relevance scores against the scale midpoint (3 = neutral). The Wilcoxon signed-rank test is the non-parametric equivalent of the one-sample t-test and was chosen to maintain methodological consistency with the other non-parametric tests employed in this study. The associated effect size is the rank-biserial correlation (r), which quantifies the proportion of observations exceeding the reference value; values of.10,.30, and.50 correspond to small, medium, and large effects [35].

To examine whether there was a hierarchy of priorities within each group, we performed separate Kruskal-Wallis tests for each subsample, with the 24 needs as the independent variable and relevance scores as the dependent variable. To assess whether the four user subsamples shared the same ordering of the 24 needs, we computed Kendall’s coefficient of concordance (W) on the mean relevance rankings across groups. Pairwise Spearman rank-order correlations between user subsamples were also calculated to identify which pair diverges most in their priority structure.

All inferential statistics were calculated with R (version 4.5.2). The dataset is available from Zenodo [36]. Supporting figures and tables complementing the one included in this paper are available from Zenodo [37].

### 2.8 Participants

Healthcare is a highly complex ecosystem encompassing various types of stakeholders, i.e., individuals and organizations that influence or are influenced by the process at stake [38]. The healthcare stakeholders mapped by previous studies [39–46] can be accommodated within three concentric circles, like in [3]. The outermost circle encompasses stakeholders who build the technical, administrative, and regulatory infrastructure for the use of AI (e.g., local and national ministries, policymakers, funding bodies and donors, health education agencies, professional orders, trade unions, and competing health facilities). An intermediate circle includes stakeholders who, although external to the health facility, interact directly with it (e.g., patients’ families, associations, and organizations for families and/or patients, counselling services, cryogenic banks, physicians’ and psychotherapists’ private practice, pharmaceutical companies, suppliers, and insurance companies). The innermost circle represents the classes of AI users relevant to our study. It consists of four groups of users within the healthcare facility: (a) patients who are hospitalized, use telemedicine devices from home, or participate in clinical trials; (b) health professionals including doctors, nurses, paramedical staff, or X-ray technicians; (c) hospital administrators and executives; (d) non-clinical hospital units such as technical department, communication with the public or advertising.

To reflect the four types of users described above, we recruited professionals with three different expertise: (a) doctors, nurses, and paramedics; (b) managers and directors; (c) workers in the IT sector. To these three subsamples, we added a fourth subsample of (d) patients (people with chronic illness or disease and no medical education). The recruitment was carried out via the online platform Prolific [47]. Participants were adults residing in the European Economic Area, where the AI Act is enforced. Balanced quotas were recruited by biological sex, with fluent knowledge of English. Respondents could not participate in the same survey multiple times, nor could they participate in more than one subsample, thanks to Prolific’s recruitment settings. Likewise, pilot participants were excluded. A total of 1079 respondents took the survey. Incomplete surveys by respondents who did not pass the attention check (11 cases), experienced some technical issue, or withdrew were removed. The final sample consisted of 813 respondents, whose sociodemographic characteristics are presented in Table 3. Half of the sample resided in Germany, Italy, Poland, or Portugal, while the rest resided in 15 other countries within the European Economic Area. The managers’ subsample was primarily composed of individuals in managerial and accounting roles, the health professionals’ subsample consisted of doctors and nurses, and the tech subsample was distributed across various information technology roles (Table B in Zenodo archive [37]).

**Table 3:** Demographic characteristics of the sample.

	N	age Mean (SD)	biol. sex		job	country of residence
			f	m		
Managers and directors	238 29.31%	35.7 (10.1)	117	121	Director (7.56%), Manager (92.44%)	20 (Germany 11.76%, Italy 12.18%, Poland 16.81%, Portugal 17.65%, other 41,6%)
Health professionals	115 14.16%	31.2 (9.3)	61	54	Doctor (62.61%), Nurse (35.65%), paramedic (1.74%)	16 (Germany 13.04%, Italy 10.43%, Poland 13.91%, Portugal 21.74%, Other 40.88%)
Patients	229 28.2%	25.9 (5.1)	114	115	–	20 (Poland 31.44%, Portugal 15.72%, other 52.84%)
IT workers	230 28.33%	33.0 (9.1)	107	123	IT (53.04%), other (46.96%)	20 (Germany 13.91%, Italy 12.17%, Poland 16.52%, Portugal 15.65%, other 41,75%)
tot	812	31.5 (9.4)	399	413		

Regarding the presence of obscure words in our simplified IFU structure, 85% of the respondents highlighted none to two words. The participants highlighting obscure words were well distributed across subsamples (admin 29%, health professionals 14%, patients 28%, and tech 28%). The words mentioned more frequently were “resilience” (*n* = 203) and “computational” (*n* = 144). The complete list is in the Supporting Information (Table C in Zenodo archive [37]).

## 3. Results

### 3.1 Needs localization

**Fig 1:**
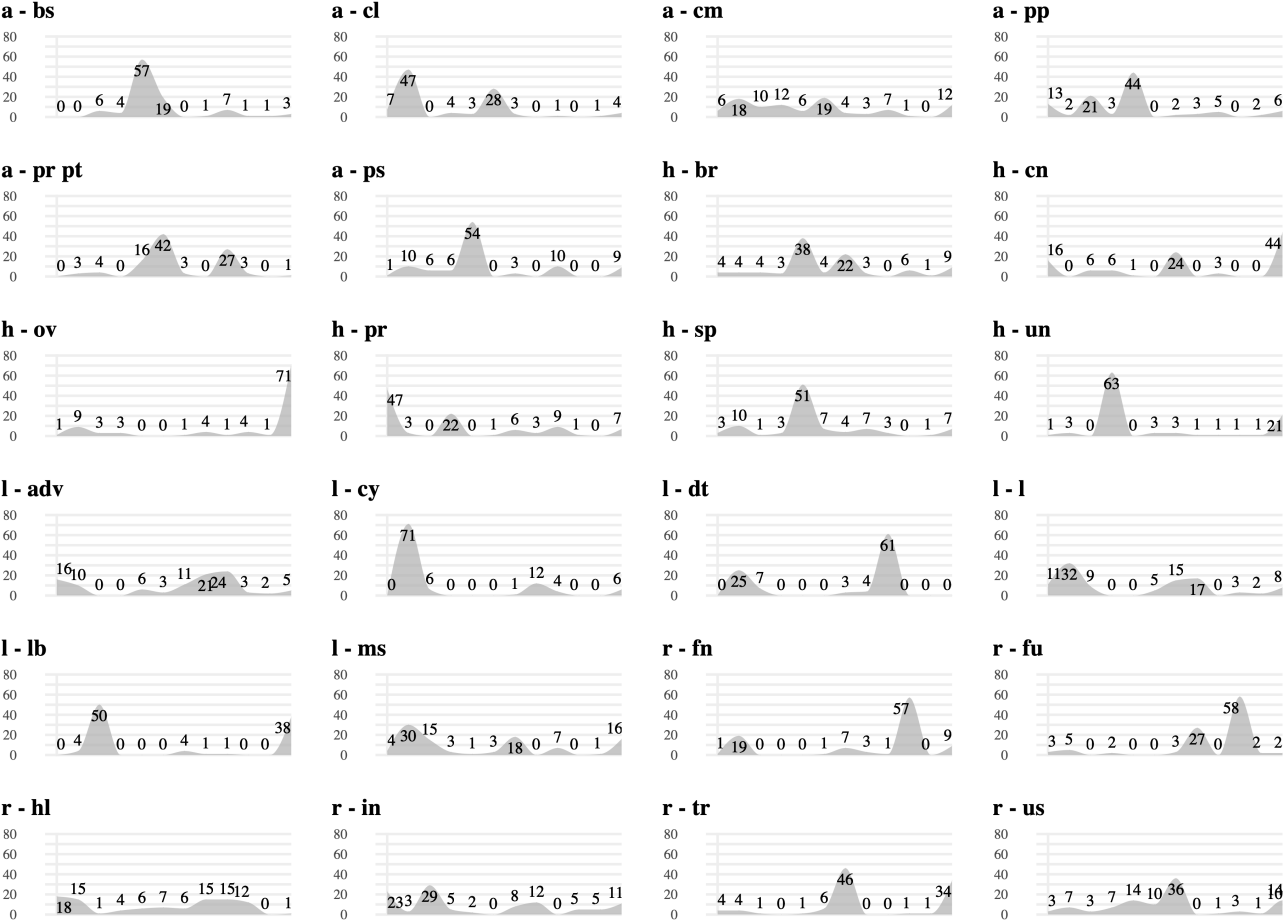
Percentage frequency of selection of each transparency need in the 12 IFU sections (x-axis of each diagram). (all diagrams were created with Flourish (*Flourish*, n.d.)).

The frequency with which each need was localized in each of the 12 IFU sections is reported in Fig 1.

Each need was presented to 63–70 participants, ensuring a sufficiently large number of observations per item (Table 4). To determine whether respondents’ choices significantly differed from a random distribution across the 12 IFU sections, we performed Chi-square tests (Table 4). If respondents had chosen at random, each of the 12 IFU sections would have received approximately 8.3% of selections (i.e., 1/12). Instead, all 24 tests were significant (p <.001, Table 4), confirming that respondents showed non-random preferences in associating transparency needs with specific IFU sections.

**Table 4:** Localizability and localization confidence of each transparency need.

Category	Transparency Need.	*N*	X^2^(11)	p	Conv. %	*M Conf.*	*SD Conf.*
***Accuracy***	a-bs	70	243.03	^***^	57	3.91	0.85
	a-cl	68	185.76	^***^	47	4.09	0.73
	a-cm	67	33.48	^***^	19	3.61	0.80
	a-pp	63	137.57	^***^	44	3.75	0.92
	a-pr pt	67	157.06	^***^	42	3.55	0.96
	a-ps	67	195.75	^***^	54	3.51	0.88
***Humanization***	h-br	68	108.12	^***^	38	3.49	0.94
	h-cn	68	163.88	^***^	44	3.57	0.95
	h-ov	69	360.04	^***^	71	4.28	0.78
	h-pr	68	167.76	^***^	47	3.96	0.80
	h-sp	67	166.37	^***^	51	3.72	0.81
	h-un	68	295.88	^***^	63	4.13	0.86
***Legal***	l-adv	63	53.76	^***^	24	2.89	1.08
	l-cy	69	367.00	^***^	71	4.38	0.69
	l-dt	69	294.65	^***^	61	4.07	0.77
	l-l	66	76.91	^***^	32	3.36	1.03
	l-lb	68	258.82	^***^	50	3.78	0.96
	l-ms	67	77.90	^***^	30	3.88	0.73
***Resources***	r-fn	68	242.24	^***^	57	3.84	0.77
	r-fu	66	259.09	^***^	58	3.30	0.93
	r-hl	68	33.65	^***^	18	3.19	0.97
	r-in	66	71.45	^***^	29	3.15	1.00
	r-tr	70	210.80	^***^	46	3.73	0.82
	r-us	70	90.80	^***^	36	3.31	0.97

N = the number of respondents who rated a given need; X^2^ = Chi-square test of IFU section selected for each need; *** = p < .001; Conv. % = percentage of respondents selecting the most frequently IFU section for each need; Conf. = mean confidence in section choice (1–5 scale).

In Fig 1, each diagram represents the distribution of a need across the 12 IFU sections. It can be observed that a few diagrams have one clear peak. Most diagrams have multiple or no peaks, and, in these cases, the highest score barely reaching 50% of the sample. This can be observed in 16 cases (a – cl, a – cm, a – pp, a pr pt, h – br, h – cn, h – pr, l – adv, l – l, l – ms, r – hl, r – in, r – tr, r – us), equaling 66% of the needs under evaluation. The distribution of a need across the 12 IFU sections is quantified by the magnitude of the Chi-square values in Table 4: higher values indicate that respondents strongly concentrated their choices on one or a few sections, whereas lower values indicate more dispersed, yet still non-random, selections. For instance, cybersecurity (l-cy, χ²(11) = 367.00) and human oversight (h-ov, χ²(11) = 360.04) showed the strongest convergence, meaning that most respondents agreed on which IFU section should contain this information. Conversely, system complexity (a-cm, χ²(11) = 33.48) and healthcare integration (r-hl, χ²(11) = 33.65) showed the most dispersed patterns, suggesting less consensus on where to locate these needs within the IFU structure, although respondents’ choices were still significantly non-random.

Table 4 also reports the mean confidence reported by participants when locating a need in the IFU structure. The Spearman correlation between confidence scores and convergence rate, i.e., the proportion of respondents selecting the most frequently chosen section, was positive and statistically significant (ρ =.695, p <.001). This suggests that needs converging more strongly on a few sections were also accompanied by higher confidence. For instance, cybersecurity (l-cy, convergence = 71%, *M* confidence = 4.38) and human oversight (h-ov, 71%, M = 4.28) showed both the highest convergence and the highest confidence, whereas healthcare integration (r-hl, 18%, M = 3.19) and advertising disclosure (l-adv, 24%, M = 2.89) showed the most dispersed selections and the lowest confidence.

### 3.2 Needs’ relevance

**Fig 2:**
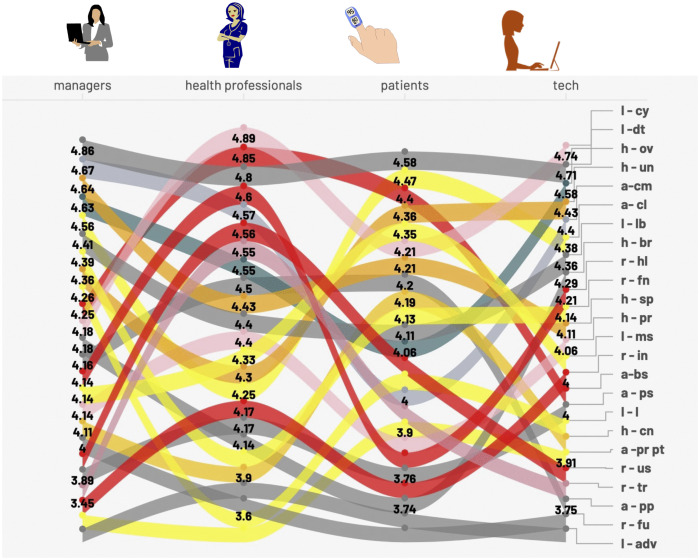
Needs’ relevance scores, ranked from the highest to the lowest in each subsample. The waved lines connect the scores of a single need across subsamples to appreciate its variation. (This diagram was created with Flourish [48]; clipart from OpenClipART [49]).

The relevance attributed by the participants to the needs included in this survey is reported in Fig 2 (and, in tabular format, in Table D in Zenodo archive [37]).

We first assessed if the needs were considered relevant by using Wilcoxon signed-rank tests to compare the relevance scores with the neutral midpoint of the scale (3 = “neither relevant nor irrelevant”). All 24 needs were rated significantly above neutral (all p <.001, Table E in Zenodo archive [37]), with large rank-biserial correlations throughout (r range: 0.54–1.00). The rank-biserial correlation r indicates the proportion of respondents whose score exceeds the neutral midpoint relative to those whose score falls below it: an r of 1.00 means that no respondent rated the need at or below neutral. The highest-rated need was data protection (l-dt, Mdn = 5, M = 4.72, r = 1.00), followed by human oversight (h-ov, Mdn = 5, M = 4.45, r = 0.95) and cybersecurity (l-cy, Mdn = 5, M = 4.43, r = 0.89). Even the lowest-rated need, advertising disclosure (l-adv, Mdn = 4, M = 3.52, r = 0.54), was rated significantly above neutral (p <.001).

A visual inspection of the lines connecting the relevance scores of the same need across user types in Fig 2 suggests that some needs seem equally ranked by the different subsamples, while other lines form steep slopes, suggesting different prioritization across subsamples. Thus, we calculated the rank position of each need in each subsample, based on their relevance scores, and the highest difference in rank across subsamples (Table 5). We first tested the statistical significance of such ranking with a within-group Kruskal-Wallis H test, treating each need as a separate group within each subsample. We found that the 24 transparency needs differed significantly in perceived relevance within each user group: health professionals, H(23) = 41.44, p =.011, ε² =.181, large effect; managers, H(23) = 64.05, p <.001, ε² =.135, medium effect; patients, H(23) = 41.70, p =.010, ε² =.091, medium effect, and tech experts, H(23) = 49.66, p =.001, ε² =.109, medium effect.

**Table 5:** Within-group rankings (1 = lowest, 24 = highest) of each transparency need across four user groups.

Transparency Need	Managers	Health Prof.	Patients	IT workers	Rank Range
	*M (Rank)*	*M (Rank)*	*M (Rank)*	*M (Rank)*	
l-dt	4.86 **(1)**	4.80 (3)	4.58 **(1)**	4.71 **(2)**	2
l-adv	3.33 **(24)**	3.88 (21)	3.67 **(23)**	3.42 **(24)**	3
a-ps	3.89 (20)	4.17 (17)	3.76 (20)	4.00 (15)	5
l-lb	4.41 (6)	4.40 (11)	4.11 (11)	4.36 (7)	5
h-pr	4.14 (16)	4.40 (12)	3.79 (18)	4.06 (12)	6
r-fu	4.00 (18)	3.73 (22)	3.61 **(24)**	3.65 **(23)**	6
a-pr pt	3.45 **(23)**	3.60 **(24)**	3.90 (17)	3.91 (19)	7
h-sp	4.64 (3)	4.43 (10)	4.21 (7)	4.11 (11)	8
r-in	3.71 (22)	4.17 (16)	3.75 (21)	4.00 (14)	8
h-ov	4.62 (4)	4.55 (8)	4.06 (12)	4.58 (3)	9
l-ms	4.14 (14)	4.33 (13)	4.35 (5)	4.05 (13)	9
h-un	4.36 (8)	4.30 (14)	4.36 (4)	4.43 (4)	10
l-cy	4.18 (11)	4.89 **(1)**	4.21 (6)	4.74 **(1)**	10
h-cn	4.11 (17)	3.90 (20)	4.19 (9)	3.93 (18)	11
r-fn	4.39 (7)	4.00 (19)	4.12 (10)	4.14 (10)	**12**
a-cl	4.56 (5)	4.25 (15)	4.47 **(2)**	4.38 (6)	**13**
a-cm	4.67 **(2)**	4.57 (5)	4.00 (15)	4.40 (5)	**13**
a-bs	4.25 (10)	4.85 **(2)**	4.40 (3)	4.00 (16)	**14**
a-pp	4.16 (13)	4.50 (9)	4.20 (8)	3.75 (22)	**14**
h-br	4.18 (12)	4.14 (18)	3.74 (22)	4.29 (8)	**14**
r-tr	3.78 (21)	4.55 (7)	3.95 (16)	3.86 (21)	**14**
r-us	3.90 (19)	4.56 (6)	4.00 (14)	3.86 (20)	**14**
l-l	4.26 (9)	3.62 **(23)**	4.00 (13)	3.93 (17)	**14**
r-hl	4.14 (15)	4.60 (4)	3.76 (19)	4.21 (9)	**15**

Rank Range indicates the maximum difference in rank position across groups; higher values signal greater divergence in prioritization. Bold rank values indicate the two highest-ranked (1–2) and two lowest-ranked (23–24) needs within each group. Bold Rank Range values (≥12) indicate needs with the largest cross-group divergence.

We then tested the extent of the agreement in the four subsamples’ ranking of the 24 needs with the Kendall’s coefficient of concordance, finding it significant, W =.647, 𝜒²(23) = 59.55, p <.001. We then improved the granularity of the comparison by running a Pairwise Spearman correlations, finding that it ranged from ρ =.40 (managers vs health professionals, p =.053) to ρ =.75 (managers vs IT workers, p <.001; Table F in Zenodo archive [37]), indicating that some user pairs prioritize needs more similarly than other, thereby accounting for the slopes in Fig 2.

## 4. Discussion

### 4.1 Design issues

The data collected in our survey shows where users localized the information related to a given transparency need. By reverse engineering this data, IFU writers can identify where to place information to maximize its localizability. For example, IFU section 2 was expected to host information about correctness, performance resilience, and cybersecurity, the evidence about the AI system’s reliability (a – cl) and its connection to the regional healthcare system (r – hl). This section could also mention which measures it incorporates to prevent intrusions from unauthorized outsiders (l – cy) and misuse or fraud (l - l). Here could also be cited the regulations and guidelines it must comply with (l – l).

The same data shows how ambiguous the mapping of transparency information onto the IFU structure is, and that, for some needs, it is hard to guess where to locate the related information. Well-mapped needs are, for example, the protection against unauthorized users (l-cy) and information on human oversight (h-ov), which 71% of the participants identified respectively with the sections “Correctness, performance resilience and cybersecurity” and “Human control over the machine.” Conversely, information about the AI device being integrated with the healthcare system of one’s region (r - hl) is distributed over five IFU sections. The localization of the transparency needs across multiple IFU sections was also accompanied by lower users’ confidence in selecting the sections, suggesting that ambiguity was not only a statistical result, but was also part of the respondent’s experience. This ambiguity would force users to spend time locating information instead of reading it, and might even result in users abandoning the search altogether.

A possible remark is that some needs are not properly part of the IFU, and this is why they are difficult to locate. However, the way the regulators modeled the IFU should not be considered as the finalized interface of the documentation set presented to the users. The designer of the documentation set, of which the IFU is part, can make room for information not explicitly prescribed by Article 13 but expected and deemed relevant by users.

Another purpose of our evaluation was to consider all the main types of deployers. The AI Act only distinguishes between developers and deployers, so managers, health professionals, patients, and IT workers are all deployers under the law. Instead, we included subsamples reflecting the different expertise of the main types of AI healthcare deployers. It might be argued that patients are not always direct users of health devices equipped with AI, and then fall outside the scope of the transparency requirements under Article 13; nevertheless, they have a right to informed consent [21]. The results suggest that all 24 transparency needs used in the study were considered relevant by the respondents, but that some were prioritized differently across user types.

### 4.2 Design recommendations

Transparency documentation designers might reduce localization ambiguities by making existing information more recognizable, including content that, while not explicitly prescribed by Article 13, is expected by users, and help navigate the documentation for people with different priorities. We suggest integrating the mandatory transparency information within a more comprehensive document, one that matches the users’ information needs. This general recommendation breaks down into five strategies:

1. ***Adding navigation interfaces***. The documentation set, of which the IFU is part, requires an additional layer to access its information according to users’ transparency needs. Gils and colleagues, based on their legal design workshops [26], recommend using a logical structure and table of contents to achieve this goal.
2. ***Diversifying access by stakeholders’ priorities.*** The documentation set can prioritize the most relevant transparency information based on users’ shared hierarchy of needs, foregrounding the most relevant information to each deployer’s type.
3. ***Locally adding case-specific information***. Several transparency needs are specific to the context in which the AI system is deployed. The provider can then include signposts for this information to be filled in locally.
4. ***Offering*** a ***reference to other documents and sources***. An IFU cannot exhaust all information needs, nor does the documentation need to be bulky and redundant; thus, the IFU can include pointers to information handled elsewhere, e.g., regulations. Gils et al [26] have a similar remark about pointers to technical manuals.
5. ***Empirically checking information localizability.*** User studies can help prepare usable documentation and support the drafting process by identifying information needs and the section most suitable to host the related information. They can also guide the assessment of a finalized draft and measure the ease with which stakeholders can locate needed information.

In addition to these specific recommendations, the IFU can follow design guidelines to make the document more readable and comprehensible, as discussed in existing literature and practical guides. For example, Crisan et al. [50] suggest that notices and cards should include warnings, prompts, and summaries, which clarify how to act on the information provided. Seifert and colleagues [51] propose labels conveying the transparency level of an AI system.

### 4.3 Limits

This study did not directly test what the participants understood about the content of the sections. This would have required a different setup with comprehension checks. Also, we did not investigate why certain words were considered obscure, which could be an interesting line of research connected to the topic of readability of transparency documentation, e.g., [52]. Managers and IT workers recruited in our sample were not explicitly employed in hospital facilities, since we were interested in the effect of their educational and professional perspectives, not in their experience with healthcare AI. Finally, the information needs highlighted by our study were very generic, as were the subsamples, to prove a concept; IFUs for specific AI technologies will need to be tested with more specific end-users.

## 5. Conclusions

It is of general societal interest that algorithmic systems are accompanied by meaningful transparency documentation. The present study shows that regulatory prescriptions do not exhaust the transparency requirements in this respect. The assessment approach we have adopted can be useful for designers of specific high-risk systems to spot needs that are opaquely reflected in their documentation and align with the sort of meaningful transparency that regulators desire. Its main components can fit different domains besides healthcare, and consist of outlining a list of user-based transparency needs, recruiting the different types of users targeted by the documentation, and asking users to validate the needs and locate them in the IFU sections.
