# Supplementary material for: Transparency in healthcare AI: Testing EU regulatory provisions against users’ transparency needs
Source: PLOS Digit Health. 2026 Jul 24;5(7):e0001594. doi: 10.1371/journal.pdig.0001594 (PMC13399356; doi:10.1371/journal.pdig.0001594)
Supplement: Attachment — Submitted filename: Response to reviewers.pdf [file pdig.0001594.s002.pdf]

Reviewer 1

Comment	Action
1. Introduction.<br>The introduction outlines a commendable overview of AI regulatory frameworks but falls short regarding the study's position within digital health implementation science and the overall context of AI regulatory usability research. The authors can further connect the study's aim to user-experience frameworks as described in the standard of ISO/IEC 62304, an established regulatory usability standard	Thank you for your remark. In the Introduction, we added references to usability<br>*The field of usable privacy [22] analyses compliance with privacy and regulatory provisions from the perspective of its usability, defined as "the extent to which a system, product or service can be used by specified users to achieve specified goals with effectiveness, efficiency and satisfaction in a specified context of use" [23]. Specifically, full compliance with the principle of transparency requires that the transparency information is understandable and relevant to users ("meaningful transparency" [24]; [25]).*<br><br>and to digital health implementation:<br>*In the area of digital health, they combine with regulations for medical devices, such as [17], [18] in the EU, to define what to include in the device's technical documentation [19] [20].*
2. Methodology and results<br><br>Although the authors mention that the data collection design employed two transparency needs per participant to avoid careless answers from fatigued participants, doesn't this introduce limitations in representativeness per need? Can the authors justify this statistically, and how did it avoid uneven response distribution? The authors can give clarity on how randomization ensured significant coverage across needs and stakeholder types.	We achieved even response distribution per need by using a dedicated feature of the survey platform Qualtrics, which allows to evenly present the randomized elements across the sample. Here is a screenshot:<br>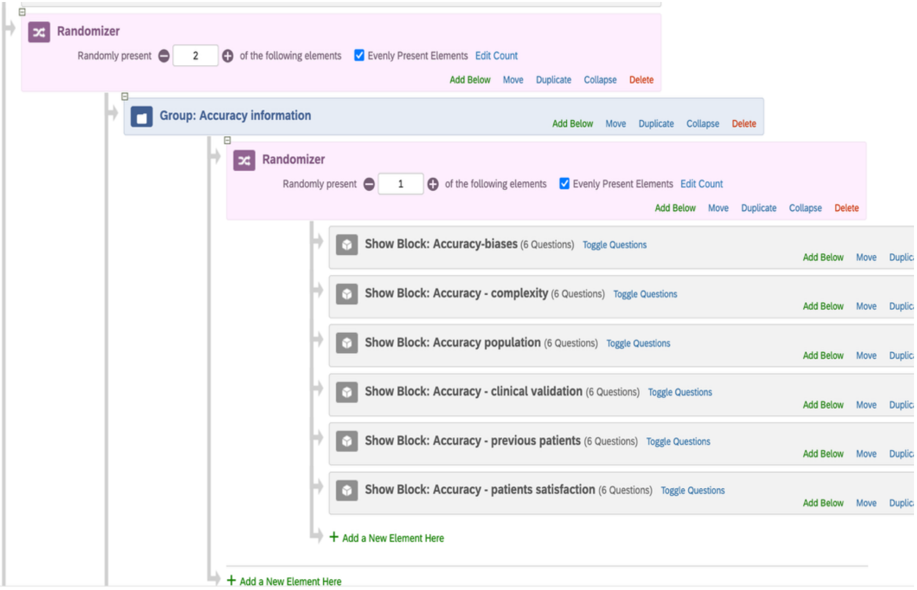<br>We referred to this feature when we wrote "The randomizers were set to display all needs the same amount of times across each subsample." We now add the words in parenthesis:

	The randomizers were set to display all needs the same number of times across each subsample (by selecting "evenly present elements" in Qualtrics randomizers).<br>Please also notice that Table 3 reports the number of participants (N) who rated each need; this is now clarified in the legend.<br>*N = the number of respondents who rated a given need*
3. The authors mention that a pilot was performed and suggestions were made. These suggestions have not been clearly stated to prove the validation of the tool used.	The only change derived from participant's suggestion was letting the instructions visible longer. This is now clarified in the Pilots section.<br>*We surveyed 12 participants to test the data collection procedure and added an item to solicit feedback. Based on these pilots, we made some adjustments to the initial setup of the survey mechanism. The change inspired by the participant's feedback consisted of autoloading all instructions within one instructions page instead of automatically progressing through several instruction pages, to accommodate every reading speed.*
4. The authors state that incomplete surveys by respondents who did not pass the attention check were among those excluded. What was the length of the attention check, and is this based on any literature that can be cited?	The attention check was one multiple choice question item. In the Procedure section we now add this clarification:<br>*An attention check was then administered and marked as such to signal the need to answer that item carefully and avoid adverse reactions observed with hidden attention checks [34]. It read as follows: "So, what is an IFU? [Answer options]: A document with instructions for the users of an AI device [correct]/an AI device supporting physicians in healthcare[wrong]/the name of a recent EU regulation regarding healthcare [wrong]."*
5. Although the authors describe explicitly the descriptive statistics, the study findings can also be further improved by providing some inferential analysis to identify statistically significant patterns	Inferential statistics were added to support all findings. In the Data Analysis section, we report the tests used. Specifically:<br>*To test if a need was univocally mapped onto a given section, we considered the frequency with which all 12 sections were chosen for that need and assessed if each section had an equal probability of being picked with a Chi-Square test. To test whether the level of convergence on the IFU sections correlated with the participants' confidence, we calculated the Spearman rank-order correlation between the proportion of respondents selecting the most frequently chosen section and the mean confidence score across the 24 transparency needs.*<br>*To validate the perceived relevance of the needs included in the study, we performed Wilcoxon signed-rank tests comparing each need's relevance scores against the scale midpoint (3 = neutral). The Wilcoxon signed-rank test is the non-parametric equivalent of the one-sample t-test and was chosen to maintain methodological consistency with the other non-parametric tests employed in this study. The*

	associated effect size is the rank-biserial correlation (r), which quantifies the proportion of observations exceeding the reference value; values of .10, .30, and .50 correspond to small, medium, and large effects [35].<br>*To examine whether there was a hierarchy of priorities within each group, we performed separate Kruskal-Wallis tests for each subsample, with the 24 needs as the independent variable and relevance scores as the dependent variable. To assess whether the four user subsamples shared the same ordering of the 24 needs, we computed Kendall's coefficient of concordance (W) on the mean relevance rankings across groups. Pairwise Spearman rank-order correlations between user subsamples were also calculated to identify which pair diverges most in their priority structure.*<br>*All inferential statistics were calculated with R (version 4.5.2).*
6. The authors have provided informative mapping results in Table 3 and Figures 2–3, but these can be difficult to<br>interpret due to the format. The authors can include a summary table or graph, or a heatmap organizing IFU sections against stakeholder clarity or confidence levels to give easier visibility.	Thank you for highlighting this.<br>About IFU sections, we better distinguished the purpose of Table 3 and Figure 2: detailed IFU sections choices are displayed in Figure 2, while Table 2 reports convergence indices and average confidence scores.<br>About need's relevance, we now offer a full breakdown per user type in Tables 4 and S4 Table.
7. Discussion and limitation<br>The authors should specify whether there were any duplicate participants and how they handled those who fell<br>into both categories of patient and doctor/nurse/paramedic, if any participated.	Thank you for giving us the chance to clarify this. The recruitment platform Prolific includes the pre-screeners called "Exclude participants from other studies." By selecting it, we excluded that the same person participated in more than one subsample. In addition, Prolific was set not to allow multiple submissions by the same user within the same subsample. So, we had no duplicate in our sample.<br>We now explain this in the Participants section:<br>*Respondents could not participate in the same survey multiple times, nor could they participate in more than one subsample, thanks to Prolific's recruitment settings. Likewise, pilot participants were excluded.*
8. Minor Comment<br>Reference formatting kept shifting in some sections between APA and	Thank you, we applied Vancouver style as recommended by PLOS Digital Health.

numeric styles; standardize according to PLOS Digital Health journal guidelines.	

Reviewer 2

Comment	Action
1. The manuscript would benefit from a brief clarification in the Discussion regarding the role of patients as deployers, particularly to distinguish between patients as direct users and as recipients of AI-supported clinical decisions.	Good point. We added the following to the Discussion: *It might be argued that patients are not always direct users of health devices equipped with AI, and then fall outside the scope of the transparency requirements under Article 13; nevertheless, they have a right to informed consent [21].*
2. While the study appropriately focuses on information localizability within the IFU structure, adding one explicit sentence in the Conclusion to distinguish localizability from actual comprehension would further strengthen the conceptual framing.	This is now acknowledged in the Limits section: *This study did not directly test what the participants understood about the content of the sections. This would have required a different setup with comprehension checks. Also, we did not investigate why certain words were considered obscure, which could be an interesting line of research connected to the topic of readability of transparency documentation, e.g., [50].*
3. Optionally, a short remark on how this evaluative approach could be extended to other high-risk AI domains under the AI Act could enhance the broader applicability of the findings.	The suggested remark is now in the Conclusions *The assessment approach we have adopted can be useful for designers of specific high-risk systems to spot needs that are opaquely reflected in their documentation and align with the sort of meaningful transparency that regulators desire. Its main components can fit different domains besides healthcare, and consist of outlining a list of user-based transparency needs, recruiting the different types of users targeted by the documentation, and asking users to validate the needs and locate them in the IFU sections.*

Reviewer 3

Comment	Action
1. The study empirically tests how participants map transparency needs to IFU section titles based on a simplified table of contents. This design primarily evaluates information architecture and	We acknowledge that insufficient coverage is a different issue than the one tacked by the study and revised the paper throughout to amend it from any confusion. The remaining reference to coverage is the following sentence in the Discussion:

navigability, not whether the IFU substantively covers deployers' transparency needs. However, parts of the Results and Discussion interpret low agreement as evidence of limited coverage by the AI Act–prescribed IFU topics.<br>This conceptual mismatch weakens the central claim. The authors should revise the framing to clearly distinguish between navigability, labeling ambiguity, and content coverage, or provide additional evidence to justify claims regarding insufficient coverage.	*A possible remark is that some needs are not properly part of the IFU, and this is why they are difficult to locate. However, the way the regulators modeled the IFU should not be considered as the finalized interface of the documentation set presented to the users. The designer of the documentation set, of which the IFU is part, can make room for information not explicitly prescribed by Article 13 but expected and deemed relevant by users.*
2. The manuscript emphasizes differences in transparency priorities across stakeholder groups (managers, healthcare professionals, patients, and IT experts). However, participants were recruited via a crowdworking platform, and there is no verification that managers and IT experts are directly involved in healthcare AI deployment contexts. Without such verification, it is difficult to interpret observed differences as deployer-role effects rather than differences in background knowledge or expectations. The authors should either moderate claims about stakeholder-specific needs or provide additional justification or controls to support these comparisons	Good point.<br>We removed the sentence "These subsamples represent the four classes of potential healthcare AI deployers we were interested in." The description of the section now reads:<br>*To reflect the four types of users described above, we recruited professionals with three different expertise: (a) doctors, nurses, and paramedics; (b) managers and directors; (c) workers in the IT sector. To these three subsamples, we added a fourth subsample of (d) patients (people with chronic illness or disease and no medical education).*<br>Also, we no longer refer to our participants as classes of "stakeholders" but of "users types." The term stakeholder is retained in more general discussion, without implying that we recruited stakeholders
3. Although the manuscript states that transparency priorities differ significantly across stakeholder groups, the analysis is largely descriptive. No inferential statistics, effect sizes, or confidence intervals are	We agree. Inferential statistics were added to support all findings. In the "Data Analysis" section we now report which tests were used, and in the Results section we report the results of the tests. Specifically:<br>*To test if a need was univocally mapped onto a given section, we considered the frequency with which all 12 sections were chosen for that need and assessed if each section had an equal probability of*

reported to support these claims. Given the centrality of stakeholder comparisons to the manuscript's contribution, appropriate statistical analyses should be conducted, or the language should be revised to avoid claims of statistical significance.	*being picked with a Chi-Square test. To test whether the level of convergence on the IFU sections correlated with the participants' confidence, we calculated the Spearman rank-order correlation between the proportion of respondents selecting the most frequently chosen section and the mean confidence score across the 24 transparency needs.*<br>*To validate the perceived relevance of the needs included in the study, we performed Wilcoxon signed-rank tests comparing each need's relevance scores against the scale midpoint (3 = neutral). The Wilcoxon signed-rank test is the non-parametric equivalent of the one-sample t-test and was chosen to maintain methodological consistency with the other non-parametric tests employed in this study. The associated effect size is the rank-biserial correlation (r), which quantifies the proportion of observations exceeding the reference value; values of .10, .30, and .50 correspond to small, medium, and large effects [35].*<br>*To examine whether there was a hierarchy of priorities within each group, we performed separate Kruskal-Wallis tests for each subsample, with the 24 needs as the independent variable and relevance scores as the dependent variable. To assess whether the four user subsamples shared the same ordering of the 24 needs, we computed Kendall's coefficient of concordance (W) on the mean relevance rankings across groups. Pairwise Spearman rank-order correlations between user subsamples were also calculated to identify which pair diverges most in their priority structure.*<br>*All inferential statistics were calculated with R (version 4.5.2).*
4. The manuscript uses multiple citation formats interchangeably (e.g., numeric references, superscript citations, and author–date formats). This inconsistency makes the manuscript difficult to read and does not comply with standard journal practices. The authors should carefully review the journal's citation guidelines and apply a single, consistent in-text citation style throughout the manuscript.	Thank you for pointing this out. We now use Vancouver uniformly for citing all kinds of sources.

6. The manuscript does not consistently adhere to the journal's required sectioning (Introduction, Methods, Results, Discussion). Some methodological details appear in the Introduction or Study sections, and interpretive statements appear in the Results. The authors should reorganize the manuscript to strictly follow the journal's prescribed structure, ensuring clear separation between background, methods, results, and interpretation.	Thank you, we fixed the discrepancy with the journal's guidelines and separated results from interpretation.
