# Supplementary material for: Transparency in healthcare AI: Testing EU regulatory provisions against users’ transparency needs
Source: PLOS Digit Health. 2026 Jul 24;5(7):e0001594. doi: 10.1371/journal.pdig.0001594 (PMC13399356; doi:10.1371/journal.pdig.0001594)
Supplement: Peer review history

# Peer review: Decision Letter 0

**DOI:** 10.1371/journal.pdig.0001594.r001

**Authors:** Dukyong Yoon

26 Dec 2025

PDIG-D-25-00511

Instructions for use to deployers in Healthcare AI:

Testing EU Regulatory Provisions against Transparency Needs

PLOS Digital Health

Dear Dr. Spagnolli,

Thank you for submitting your manuscript to PLOS Digital Health. After careful consideration, we feel that it has merit but does not fully meet PLOS Digital Health's publication criteria as it currently stands. Therefore, we invite you to submit a revised version of the manuscript that addresses the points raised during the review process.

Please submit your revised manuscript by Feb 24 2026 11:59PM. If you will need more time than this to complete your revisions, please reply to this message or contact the journal office at digitalhealth@plos.org. When you're ready to submit your revision, log on to https://www.editorialmanager.com/pdig/ and select the 'Submissions Needing Revision' folder to locate your manuscript file.

Please include the following items when submitting your revised manuscript:

* A letter that responds to each point raised by the editor and reviewer(s). You should upload this letter as a separate file labeled 'Response to Reviewers'. This file does not need to include responses to any formatting updates and technical items listed in the 'Journal Requirements' section below.

* A marked-up copy of your manuscript that highlights changes made to the original version. You should upload this as a separate file labeled 'Revised Manuscript with Track Changes'.

* An unmarked version of your revised paper without tracked changes. You should upload this as a separate file labeled 'Manuscript'.

If you would like to make changes to your financial disclosure, competing interests statement, or data availability statement, please make these updates within the submission form at the time of resubmission. Guidelines for resubmitting your figure files are available below the reviewer comments at the end of this letter.

We look forward to receiving your revised manuscript.

Kind regards,

Dukyong Yoon

Section Editor

PLOS Digital Health

Dukyong Yoon

Section Editor

PLOS Digital Health

Leo Anthony Celi

Editor-in-Chief

PLOS Digital Health

orcid.org/0000-0001-6712-6626

**Journal Requirements:**

1. We ask that a manuscript source file is provided at Revision. Please upload your manuscript file as a .doc, .docx, .rtf or .tex.

2. Please insert an Ethics Statement at the beginning of your Methods section, under a subheading 'Ethics Statement'. It must include:

1) The name(s) of the Institutional Review Board(s) or Ethics Committee(s)

2) The approval number(s), or a statement that approval was granted by the named board(s)

3) (for human participants/donors) - A statement that formal consent was obtained (must state whether verbal/written) OR the reason consent was not obtained (e.g. anonymity). NOTE: If child participants, the statement must declare that formal consent was obtained from the parent/guardian.

3. Please provide an Author Summary. This should appear in your manuscript between the Abstract (if applicable) and the Introduction, and should be 150–200 words long. The aim should be to make your findings accessible to a wide audience that includes both scientists and non-scientists. Sample summaries can be found on our website under Submission Guidelines:

https://journals.plos.org/digitalhealth/s/submission-guidelines#loc-parts-of-a-submission

4. Your manuscript is missing the following sections: Method. Please ensure these are present, and in the correct order, and that any references to subheadings in your main text are correct. An outline of the required sections can be consulted in our submission guidelines here:

https://journals.plos.org/digitalhealth/s/submission-guidelines#loc-parts-of-a-submission

5. Please provide separate figure files in .tif or .eps format.

For more information about figure files please see our guidelines:

https://journals.plos.org/digitalhealth/s/figures

https://journals.plos.org/digitalhealth/s/figures#loc-file-requirements

6. Some material included in your submission may be copyrighted. According to PLOS’s copyright policy, authors who use figures or other material (e.g., graphics, clipart, maps) from another author or copyright holder must demonstrate or obtain permission to publish this material under the Creative Commons Attribution 4.0 International (CC BY 4.0) License used by PLOS journals. Please closely review the details of PLOS’s copyright requirements here: PLOS Licenses and Copyright. If you need to request permissions from a copyright holder, you may use PLOS's Copyright Content Permission form.

Please respond directly to this email or email the journal office and provide any known details concerning your material's license terms and permissions required for reuse, even if you have not yet obtained copyright permissions or are unsure of your material's copyright compatibility.

Potential Copyright Issues:

Figure 1, 3, Table 4: Please confirm whether you drew the images / clip-art within the figure panels by hand. If you did not draw the images, please provide (a) a link to the source of the images or icons and their license / terms of use; or (b) written permission from the copyright holder to publish the images or icons under our CC-BY 4.0 license. Alternatively, you may replace the images with open source alternatives. See these open source resources you may use to replace images / clip-art:

- https://commons.wikimedia.org

- https://openclipart.org/

If the reviewer comments include a recommendation to cite specific previously published works, please review and evaluate these publications to determine whether they are relevant and should be cited. There is no requirement to cite these works unless the editor has indicated otherwise.

[Note: HTML markup is below. Please do not edit.]

**Reviewers' Comments:**

Reviewer's Responses to Questions

**Comments to the Author**

1. Does this manuscript meet PLOS Digital Health’s publication criteria? Is the manuscript technically sound, and do the data support the conclusions? The manuscript must describe methodologically and ethically rigorous research with conclusions that are appropriately drawn based on the data presented.

Reviewer #1: Partly

Reviewer #2: Yes

Reviewer #3: Yes

**********

2. Has the statistical analysis been performed appropriately and rigorously?

Reviewer #1: Yes

Reviewer #2: Yes

Reviewer #3: No

**********

3. Have the authors made all data underlying the findings in their manuscript fully available (please refer to the Data Availability Statement at the start of the manuscript PDF file)?

The PLOS Data policy requires authors to make all data underlying the findings described in their manuscript fully available without restriction, with rare exception. The data should be provided as part of the manuscript or its supporting information, or deposited to a public repository. For example, in addition to summary statistics, the data points behind means, medians and variance measures should be available. If there are restrictions on publicly sharing data—e.g. participant privacy or use of data from a third party—those must be specified.

Reviewer #1: Yes

Reviewer #2: Yes

Reviewer #3: Yes

**********

4. Is the manuscript presented in an intelligible fashion and written in standard English?

PLOS Digital Health does not copyedit accepted manuscripts, so the language in submitted articles must be clear, correct, and unambiguous. Any typographical or grammatical errors should be corrected at revision, so please note any specific errors here.

Reviewer #1: Yes

Reviewer #2: Yes

Reviewer #3: Yes

**********

5. Review Comments to the Author

Please use the space provided to explain your answers to the questions above. You may also include additional comments for the author, including concerns about dual publication, research ethics, or publication ethics. (Please upload your review as an attachment if it exceeds 20,000 characters)

Reviewer #1: Congratulations to the authors for this exhaustive study. The study offers unique and policy-relevant contributions in digital health and connectivity solutions. The comments below can help in improving this writing.

Major Comments

Introduction

The introduction outlines a commendable overview of AI regulatory frameworks but falls short regarding the study’s position within digital health implementation science and the overall context of AI regulatory usability research. The authors can further connect the study’s aim to user-experience frameworks as described in the standard of ISO/IEC 62304, an established regulatory usability standard

Methodology and results

Although the authors mention that the data collection design employed two transparency needs per participant to avoid careless answers from fatigued participants, doesn’t this introduce limitations in representativeness per need? Can the authors justify this statistically, and how did it avoid uneven response distribution? The authors can give clarity on how randomization ensured significant coverage across needs and stakeholder types.

The authors mention that a pilot was performed and suggestions were made. These suggestions have not been clearly stated to prove the validation of the tool used.

The authors state that incomplete surveys by respondents who did not pass the attention check were among those excluded. What was the length of the attention check, and is this based on any literature that can be cited?

Although the authors describe explicitly the descriptive statistics, the study findings can also be further improved by providing some inferential analysis to identify statistically significant patterns

The authors have provided informative mapping results in Table 3 and Figures 2–3, but these can be difficult to interpret due to the format. The authors can include a summary table or graph, or a heatmap organizing IFU sections against stakeholder clarity or confidence levels to give easier visibility.

Discussion and limitation

The authors should specify whether there were any duplicate participants and how they handled those who fell into both categories of patient and doctor/nurse/paramedic, if any participated.

Minor Comment

Reference formatting kept shifting in some sections between APA and numeric styles; standardize according to PLOS Digital Health journal guidelines.

Reviewer #2: This manuscript presents a timely and well-executed empirical evaluation of whether the Instructions for Use (IFU) prescribed by the EU AI Act meaningfully address deployers’ transparency needs in healthcare AI. The study is methodologically sound, clearly reported, and highly relevant to the scope of PLOS Digital Health. The conclusions are well supported by the data and provide actionable insights for regulators, AI providers, and designers.

I recommend minor revisions only, aimed at further improving conceptual clarity rather than addressing any methodological limitations.

Minor suggestions:

The manuscript would benefit from a brief clarification in the Discussion regarding the role of patients as deployers, particularly to distinguish between patients as direct users and as recipients of AI-supported clinical decisions.

While the study appropriately focuses on information localizability within the IFU structure, adding one explicit sentence in the Conclusion to distinguish localizability from actual comprehension would further strengthen the conceptual framing.

Optionally, a short remark on how this evaluative approach could be extended to other high-risk AI domains under the AI Act could enhance the broader applicability of the findings.

These are minor clarifications that do not affect the validity of the results or the overall contribution of the manuscript.

Reviewer #3: This manuscript addresses a timely and important issue: whether the structure of the Instructions for Use (IFU) mandated by Article 13 of the EU AI Act meaningfully supports transparency needs in healthcare AI. The topic is highly relevant, the sample size is substantial, and the cross-stakeholder perspective is valuable. However, several conceptual, methodological, and formatting issues currently limit the strength and clarity of the conclusions. Substantial revisions are required before the manuscript can be considered for publication.

1. The study empirically tests how participants map transparency needs to IFU section titles based on a simplified table of contents. This design primarily evaluates information architecture and navigability, not whether the IFU substantively covers deployers’ transparency needs. However, parts of the Results and Discussion interpret low agreement as evidence of limited coverage by the AI Act–prescribed IFU topics.

This conceptual mismatch weakens the central claim. The authors should revise the framing to clearly distinguish between navigability, labeling ambiguity, and content coverage, or provide additional evidence to justify claims regarding insufficient coverage.

2.The manuscript emphasizes differences in transparency priorities across stakeholder groups (managers, healthcare professionals, patients, and IT experts). However, participants were recruited via a crowdworking platform, and there is no verification that managers and IT experts are directly involved in healthcare AI deployment contexts. Without such verification, it is difficult to interpret observed differences as deployer-role effects rather than differences in background knowledge or expectations. The authors should either moderate claims about stakeholder-specific needs or provide additional justification or controls to support these comparisons.

3. Although the manuscript states that transparency priorities differ significantly across stakeholder groups, the analysis is largely descriptive. No inferential statistics, effect sizes, or confidence intervals are reported to support these claims. Given the centrality of stakeholder comparisons to the manuscript’s contribution, appropriate statistical analyses should be conducted, or the language should be revised to avoid claims of statistical significance.

4. The manuscript uses multiple citation formats interchangeably (e.g., numeric references, superscript citations, and author–date formats). This inconsistency makes the manuscript difficult to read and does not comply with standard journal practices. The authors should carefully review the journal’s citation guidelines and apply a single, consistent in-text citation style throughout the manuscript.

5. The manuscript does not consistently adhere to the journal’s required sectioning (Introduction, Methods, Results, Discussion). Some methodological details appear in the Introduction or Study sections, and interpretive statements appear in the Results. The authors should reorganize the manuscript to strictly follow the journal’s prescribed structure, ensuring clear separation between background, methods, results, and interpretation.

**********

6. PLOS authors have the option to publish the peer review history of their article (what does this mean?). If published, this will include your full peer review and any attached files.

**Do you want your identity to be public for this peer review?**  If you choose “no”, your identity will remain anonymous but your review may still be made public.

For information about this choice, including consent withdrawal, please see our Privacy Policy.

Reviewer #1: **Yes:**  kabugo joel

Reviewer #2: No

Reviewer #3: No

**********

[NOTE: If reviewer comments were submitted as an attachment file, they will be attached to this email and accessible via the submission site. Please log into your account, locate the manuscript record, and check for the action link "View Attachments". If this link does not appear, there are no attachment files.]

**Figure resubmission:**

While revising your submission, we strongly recommend that you use PLOS’s NAAS tool (https://ngplosjournals.pagemajik.ai/artanalysis) to test your figure files. NAAS can convert your figure files to the TIFF file type and meet basic requirements (such as print size, resolution), or provide you with a report on issues that do not meet our requirements and that NAAS cannot fix.

After uploading your figures to PLOS’s NAAS tool - https://ngplosjournals.pagemajik.ai/artanalysis, NAAS will process the files provided and display the results in the "Uploaded Files" section of the page as the processing is complete. If the uploaded figures meet our requirements (or NAAS is able to fix the files to meet our requirements), the figure will be marked as "fixed" above. If NAAS is unable to fix the files, a red "failed" label will appear above. When NAAS has confirmed that the figure files meet our requirements, please download the file via the download option, and include these NAAS processed figure files when submitting your revised manuscript.

**Reproducibility:**

To enhance the reproducibility of your results, we recommend that authors of applicable studies deposit laboratory protocols in protocols.io, where a protocol can be assigned its own identifier (DOI) such that it can be cited independently in the future. Additionally, PLOS ONE offers an option to publish peer-reviewed clinical study protocols. Read more information on sharing protocols at https://plos.org/protocols?utm_medium=editorial-email&utm_source=authorletters&utm_campaign=protocols

# Author comment: Author response to Decision Letter 1

**DOI:** 10.1371/journal.pdig.0001594.r002

16 Apr 2026

Attachment Submitted filename: Response to reviewers.pdf

# Peer review: Decision Letter 1

**DOI:** 10.1371/journal.pdig.0001594.r003

**Authors:** Dukyong Yoon

6 Jul 2026

Transparency in Healthcare AI: Testing EU Regulatory Provisions against Users’ Transparency Needs

PDIG-D-25-00511R1

Dear Prof Spagnolli,

We are pleased to inform you that your manuscript 'Transparency in Healthcare AI: Testing EU Regulatory Provisions against Users’ Transparency Needs' has been provisionally accepted for publication in PLOS Digital Health.

Before your manuscript can be formally accepted you will need to complete some formatting changes, which you will receive in a follow-up email from a member of our team.

Please note that your manuscript will not be scheduled for publication until you have made the required changes, so a swift response is appreciated.

IMPORTANT: The editorial review process is now complete. PLOS will only permit corrections to spelling, formatting or significant scientific errors from this point onwards. Requests for major changes, or any which affect the scientific understanding of your work, will cause delays to the publication date of your manuscript.

If your institution or institutions have a press office, please notify them about your upcoming paper to help maximize its impact. If they'll be preparing press materials, please inform our press team as soon as possible -- no later than 48 hours after receiving the formal acceptance. Your manuscript will remain under strict press embargo until 2 pm Eastern Time on the date of publication. For more information, please contact digitalhealth@plos.org.

Thank you again for supporting Open Access publishing; we are looking forward to publishing your work in PLOS Digital Health.

Best regards,

Dukyong Yoon

Section Editor

PLOS Digital Health

***********************************************************

**Additional Editor Comments (if provided):**

**Reviewer Comments (if any, and for reference):**

Reviewer's Responses to Questions

**Comments to the Author**

1. If the authors have adequately addressed your comments raised in a previous round of review and you feel that this manuscript is now acceptable for publication, you may indicate that here to bypass the “Comments to the Author” section, enter your conflict of interest statement in the “Confidential to Editor” section, and submit your "Accept" recommendation.

Reviewer #1: All comments have been addressed

Reviewer #2: All comments have been addressed

**********

2. Does this manuscript meet PLOS Digital Health’s publication criteria? Is the manuscript technically sound, and do the data support the conclusions? The manuscript must describe methodologically and ethically rigorous research with conclusions that are appropriately drawn based on the data presented.

Reviewer #1: Yes

Reviewer #2: Yes

**********

3. Has the statistical analysis been performed appropriately and rigorously?

Reviewer #1: Yes

Reviewer #2: Yes

**********

4. Have the authors made all data underlying the findings in their manuscript fully available (please refer to the Data Availability Statement at the start of the manuscript PDF file)?

The PLOS Data policy requires authors to make all data underlying the findings described in their manuscript fully available without restriction, with rare exception. The data should be provided as part of the manuscript or its supporting information, or deposited to a public repository. For example, in addition to summary statistics, the data points behind means, medians and variance measures should be available. If there are restrictions on publicly sharing data—e.g. participant privacy or use of data from a third party—those must be specified.

Reviewer #1: Yes

Reviewer #2: Yes

**********

5. Is the manuscript presented in an intelligible fashion and written in standard English?

PLOS Digital Health does not copyedit accepted manuscripts, so the language in submitted articles must be clear, correct, and unambiguous. Any typographical or grammatical errors should be corrected at revision, so please note any specific errors here.

Reviewer #1: Yes

Reviewer #2: Yes

**********

6. Review Comments to the Author

Please use the space provided to explain your answers to the questions above. You may also include additional comments for the author, including concerns about dual publication, research ethics, or publication ethics. (Please upload your review as an attachment if it exceeds 20,000 characters)

Reviewer #1: (No Response)

Reviewer #2: The authors have adequately addressed the concerns raised during the previous round of review. The revised manuscript has been substantially improved, particularly through the clearer positioning of the study within healthcare AI transparency and usability research, the clarification of the survey methodology, and the addition of appropriate inferential statistical analyses.

The study addresses an important and timely topic by examining the alignment between regulatory transparency requirements under the EU AI Act and the transparency needs of different healthcare AI stakeholders. The distinction between information localizability and actual comprehension has been better clarified in the revised version.

I appreciate the authors’ efforts to moderate the interpretation of the findings and to acknowledge the limitations related to stakeholder representation and IFU evaluation. Future research may further investigate how transparency needs vary across specific types of healthcare AI systems, such as diagnostic AI, clinical decision support systems, predictive models, and generative AI applications.

Overall, the manuscript is methodologically improved, clearly presented, and provides useful insights for developing user-centered transparency documentation for healthcare AI deployment.

**********

7. PLOS authors have the option to publish the peer review history of their article (what does this mean?). If published, this will include your full peer review and any attached files.

**Do you want your identity to be public for this peer review?**  If you choose “no”, your identity will remain anonymous but your review may still be made public.

For information about this choice, including consent withdrawal, please see our Privacy Policy.

Reviewer #1: **Yes:**  kabugo joel

Reviewer #2: No

**********
